# Endogenous TDP-43 mislocalization in a novel knock-in mouse model reveals DNA repair impairment, inflammation, and neuronal senescence

**DOI:** 10.21203/rs.3.rs-3879966/v2

**Published:** 2024-03-20

**Authors:** Joy Mitra, Prakash Dharmalingam, Manohar Kodavati, Erika N. Guerrero, K. S. Rao, Ralph M. Garruto, Muralidhar L. Hegde

**Affiliations:** 1 Division of DNA Repair Research, Center for Neuroregeneration, Department of Neurosurgery, Houston Methodist Research Institute, Houston, TX 77030, USA; 2 Department of Biotechnology, Koneru Lakshmaiah Education Foundation Deemed to be University, Green Fields, Vaddeswaram, Andhra Pradesh 522502, India; 3 Department of Anthropology, Binghamton University, State University of New York, Binghamton, NY 13902, USA; 4 Department of Biological Sciences, Binghamton University, State University of New York, Binghamton, NY 13902, USA; 5 Department of Neuroscience, Weill Cornell Medical College, New York, NY 10065, USA

**Keywords:** amyotrophic lateral sclerosis, TDP-43, inflammation, DNA double-strand break, senescence, motor deficits, muscle atrophy, neurodegeneration, motor neuron

## Abstract

TDP-43 mislocalization and aggregation are key pathological features of motor neuron diseases (MND) including amyotrophic lateral sclerosis (ALS) and frontotemporal dementia (FTD). However, transgenic hTDP-43 WT or ΔNLS-overexpression animal models mainly capture late-stages TDP-43 proteinopathy, and do not provide a complete understanding of early motor neuron-specific pathology during pre-symptomatic phases. We have now addressed this shortcoming by generating a new endogenous knock-in (KI) mouse model using a combination of CRISPR/Cas9 and FLEX Cre-switch strategy for the conditional expression of a mislocalized Tdp-43ΔNLS variant of mouse Tdp-43. This variant is either expressed conditionally in whole mice or specifically in the motor neurons. The mice exhibit loss of nuclear Tdp-43 concomitant with its cytosolic accumulation and aggregation in targeted cells, leading to increased DNA double-strand breaks (DSBs), signs of inflammation and DNA damage-associated cellular senescence. Notably, unlike WT Tdp43 which functionally interacts with Xrcc4 and DNA Ligase 4, the key DSB repair proteins in the non-homologous end-joining (NHEJ) pathway, the Tdp-43ΔNLS mutant sequesters them into cytosolic aggregates, exacerbating neuronal damage in mice brain. The mutant mice also exhibit myogenic degeneration in limb muscles and distinct motor deficits, consistent with the characteristics of MND. Our findings reveal progressive degenerative mechanisms in motor neurons expressing endogenous Tdp-43ΔNLS mutant, independent of TDP-43 overexpression or other confounding etiological factors. Thus, this unique Tdp-43 KI mouse model, which displays key molecular and phenotypic features of Tdp-43 proteinopathy, offers a significant opportunity to further characterize the early-stage progression of MND and also opens avenues for developing DNA repair-targeted approaches for treating TDP-43 pathology-linked neurodegenerative diseases.

## Introduction

Neuronal degeneration in the central nervous system (CNS) linked to TAR DNA-binding protein of 43kD (TDP-43) pathology is a prominent hallmark of several neurodegenerative diseases, including amyotrophic lateral sclerosis (ALS) [1] and frontotemporal degeneration (FTD) [2]. TDP-43 pathology also features in nearly half of Alzheimer’s disease (AD) cases [3-6]. Classic ALS and FTD pathologies are distinguished by TDP-43 inclusions that are ubiquitin-positive but tau-negative [7]. Several dozen missense mutations, including both familial and sporadic, have been identified in TDP-43, primarily in the disordered prion-like domain (PLD) located at the C-terminus [8, 9]. Two consistent phenomena observed in TDP-43 pathology-linked ALS and FTD patients’ CNS tissues are the nuclear clearance and cytosolic buildup of pathogenic TDP-43 forms. The C-terminal domain (CTD) of TDP-43, while not fully characterized, was shown to interact with various cellular protein complexes, including heteronuclear ribonucleoproteins (hnRNPs), involved in a variety of cellular processes [10].

Progressive accumulation of genome damage and neuroinflammation has been consistently observed in various ALS/FTD disease models as well as in patients’ brain and spinal cord specimens [11-17]. Studies, including ours, reveal that TDP-43 plays a crucial role in executing the non-homologous end-joining (NHEJ)-mediated DNA double-strand break (DSB) repair and in resolving R-loops, both of which are implied in ALS/FTD pathology [11, 18-21]. In this context, TDP-43’s bipartite nuclear localization (NLS) and nuclear export (NES) signal sequences in association with other accessory proteins such as importin-α1/β [22], play pivotal roles in maintaining homeostasis of the TDP-43 protein content across various cellular compartments [23, 24]. Notably, the TDP-43 A90V mutation located within the NLS has been identified in familial ALS/FTD [25]. Thus, altering the NLS could recapitulate the pathology of TDP-43’s nuclear clearance, potentially mirroring ALS/FTD phenotypes in animal models.

While various TDP-43 NLS mice models have been developed to date, each has specific technical limitations in closely mirroring the patient pathology. The majority of these transgenic models include the cytoplasmic aggregates of TDP-43 for pathological signaling, but not the loss of its nuclear function. For instance, the transgenic Tet-OFF-CamkIIa-hTDP-43-ΔNLS model [26] may not reproduce the effects of endogenous Tdp-43 proteinopathy at the molecular level due to variable interaction and RNA processing abilities of human TDP-43-WT or −ΔNLS in the presence of background murine Tdp-43. Moreover, this model lacks the ability to induce TDP-43 proteinopathy in the spinal cord. Another Tet-OFF-NEFH-hTDP-43-ΔNLS model that induces TDP-43 aggregation in both the brain and spinal cord in order to study the impact on murine Tdp-43 [27] shown similar limitations. A third model, which completely deletes the Exon3 containing the Tdp-43 NLS sequence, likely introduces non-NLS-related cellular anomalies due to the loss of RNA recognition motif (RRM) along with the NLS [28, 29]. Thus, there is an urgent need for a suitable *Tardbp* mouse model that can delve into the progression of Tdp-43 toxicity in specific developmental stages or tissue/cell types, which is vital for understanding ALS/FTD's pre- to post-symptomatic transition.

To confront challenges of studying age-associated progression of ALS and deciphering the pathological cellular modifications at the onset or pre-symptomatic stages, we developed a new endogenous conditionally expressing Tdp-43ΔNLS (Δ82-98 aa) mouse model of ALS. This model allows us to initiate Tdp-43ΔNLS expression endogenously in any desired cell/tissue type and developmental stage, simply by cross-breeding the Tdp-43ΔNLS strain with an appropriate Cre-expressing mouse strain. In this study, we demonstrate that this model effectively captures the key pathological hallmarks of ALS, including TDP-43 aggregation, genomic instability, neuroinflammation, and neuronal senescence, while offering a promising approach for exploring the onset of DNA repair impairment and its associated pathology in ALS/FTD. Furthermore, this model provides a foundation for exploring early-stage ALS/FTD treatment strategies.

## Materials and methods

### Cell culture and treatments

Human neuroblastoma SH-SY5Y cells (ATCC, #CRL-2266) were cultured in DMEM/F12 supplemented with 10% fetal bovine serum (Sigma) and 1% penicillin-streptomycin (Gibco) at 37 °C with 5% CO_2_. SH-SY5Ycells were differentiated in 10 μM retinoic acid (RA) and 50 ng/mL BDNF in DMEM/F12 with 1% FBS for 5 days [11]. Doxycycline (Dox)-inducible TDP-43 WT and NLS mutated (mNLS) SH-SY5Y lines were generated by transfecting pCW-TDP-43 plasmids using Lipofectamine-3000 (Life Technologies) and selecting against puromycin (Invivogen). For TDP-43 expression, Dox was given at 5 μg/mL final concentration for 72 h under the differentiated condition. TDP-43 downregulation was achieved by RNA interference to TDP-43 (siTDP-43) as described elsewhere [11].

### Comet assay

The neutral comet assay was performed using the Comet Assay Kit (Trevigen, #4250-50-K), according to the manufacturer’s protocol to assess the extent of DNA DSBs in each sample. Briefly, singlet cell suspension was prepared by trypsinization of control and induced SH-SY5Y cells in DPBS buffer and about 200 cells were smeared in LMAgarose at 37 °C in duplicate in each slide. The comet tails were visualized by staining the DNA with SYBR Green gold stain under a fluorescence microscope.

### Western blotting (WB)

Cells harvested, pelleted at 1,500 rpm at 4 °C for 5 min and lysed with whole cell lysis buffer (50 mM Tris-HCl pH 7.5, 150 mM NaCl, 1 mM EDTA, and cocktail protease inhibitors). Protein concentration was determined using the Bradford assay (Sigma). Protein bands were separated on a NuPAGE 4-12% Bis-Tris Gel (Invitrogen). Proteins were electrotransferred onto a nitrocellulose membrane in 1X NuPAGE transfer buffer. After blocking with 5% skimmed milk solution in 1% Tris-Buffered saline with Tween 20 (TBST) buffer, the membranes were immunoblotted with mouse anti-Flag antibody (Sigma, #F3165, 1:1000), rabbit anti-TDP-43 (Proteintech, #10782-2-AP, 1:1000), mouse anti-phospho-Histone H2.AX (S139) (EMD Millipore, #16-193, 1:1000), rabbit anti-H2.AX (Cell Signaling Tech, #2595, 1:1000), rabbit anti-phospho-ATM (S1981) (Abcam, #ab81292, 1:800), rabbit anti-ATM (Abcam, #ab32420, 1:1000), rabbit anti-phospho-53BP1 (S1778) (Cell Signaling Tech, #2675, 1:1000), rabbit anti-53BP1 (Cell Signaling Tech, #4937, 1:1000), and mouse anti-β-actin (Proteintech, #66009-1-Ig, 1:5000) antibodies. Protein bands were visualized by probing with corresponding HRP-conjugated secondary antibodies and developed with enhanced chemiluminescence reagent in Odyssey (LI-COR).

### Cell viability (MTT) assay

TDP-43 WT or mNLS expressing SH-SY5Y cells exposed to sodium arsenite (NaAs) at 0.5 mM final concentration for 30 min in a 96-well ELISA plate (Corning) in triplicates and recovered at 0, 6, 12, and 24 h timepoints [30]. MTT assay was performed following the manufacturer’s protocol (Trevigen). Briefly, 10 μL of MTT reagent was added to each well and incubated for 2-4 h until purple dye was visible, then 100 μL of detergent reagent was added. After 2 h of incubation, absorbance at 570 nm was measured in a microplate reader (Bio-Rad, 680 XR).

### Generation of Tdp-43ΔNLS and bigenic Cre::Tdp-43ΔNLS mice

The endogenous Tdp-43ΔNLS mice were generated by injecting a linearized plasmid carrying murine *Tardbp* NLS-deleted Exon3 (mExon3) in the reverse orientation following the WT Exon3 and flanked by pairs of WT and mutant loxP sequences with a 5'- (intronic region of ~2 kb between Exon2 and Exon3) and 3'- (~2 kb region downstream of Exon3) homology arms in the background of C57BL/6. To induce the recombination process, two DNA DSBs were introduced flanking the WT Exon3 by CRISPR/Cas9 technique. The F1 founder line was screened by a standard genotyping PCR using the primer pairs: loxP-Tdp-F 5'-AAAACACTTGCAGAGCAAGCCTGAC-3' and loxP-Tdp-R 5'-TGGTTGGAGTGATTTTTCTAGTACCCCC-3' in a touchdown PCR protocol (denaturation at 94 °C for 5 min; 94 °C – 30 sec, 67 °C – 30 sec, 68 °C – 30 sec for 15 cycles; 94 °C – 30 sec, 57 °C – 30 sec, 68 °C – 30 sec for 25 cycles; final elongation at 68 °C – 10 min). The founder line was maintained by backcrossing with non-carrier C57BL/6 mice. Four original founder lines were produced, however, only one hemizygous line was used in this study. Other lines were cryopreserved as backup. Next, the bigenic Cre::Tdp-43ΔNLS mice were generated by crossing the monogenic Tdp-43ΔNLS line with either tamoxifen (TAM)-inducible Ubc-Cre-ERT2 transgenic line (#008085, The Jackson Laboratory) or Mnx1-Cre line (#006600, The Jackson Laboratory) to establish the whole-body or motor neuron (MN)-specific Tdp-43ΔNLS mouse line. The Cre expression under Ubc promoter was induced by intraperitoneally injecting 75 mg/kg of TAM (#T5648; Sigma) or corn oil (vehicle) every other day for 2 consecutive weeks [31].

Genotyping was performed using earpiece DNA as described previously [32]. All mice were housed in ventilated microbarrier cages on racks providing high efficiency particulate air (HEPA)-filtered air supply to each cage. Animals were kept on a 12-h light–dark cycle with *ad libitum* access to food and water. All animal husbandry, experiments, and procedures were performed in strict compliance with animal protocols in accordance with the NIH Guide for the Care and Use of Experimental Animals and approved by the Institutional Animal Care and Use Committee (IACUC) of the Houston Methodist Research Institute (Protocol # IS00006797) as well as following the current laws for laboratory animal care and handling of the United States.

### Immunohistochemistry (IHC)

Mice brain, spinal cord, and soleus muscle tissues were immediately harvested after anesthesia with 30% of Isoflurane, and half of the tissue samples were snap-frozen in liquid nitrogen for genetic and biochemical analysis, while the other half were stored in 4% paraformaldehyde (PFA) in 0.1 M phosphate buffer for IHC and IF analysis. Tissue samples were paraffin embedded and sliced into 5 μm horizontal sections and mounted on glass slides. Slides were dewaxed and autoclaved for 10 min at 121°C in 0.01 M citrate buffer pH6.0 for antigen retrieval. Immunostaining was performed using overnight incubation at 4 °C with mouse anti-TDP-43 (R&D Systems, #MAB77781, 1:200), rabbit anti-TDP-43 (Proteintech, #10782-2-AP, 1:250), rabbit anti-phospho-TDP-43 (S409/410) (Proteintech, #80007-1-RR, 1:300), anti-phospho-Histone H2.AX (S139) (Abcam, #ab81299, 1:200), mouse anti-Neuronal Nuclei (NeuN) (Proteintech, #66836-1-Ig, 1:100), mouse anti-Glial Fibrillary Acidic Protein (GFAP) (Proteintech, #60190-1-Ig, 1:100), mouse anti-Iba1 (Sigma, #SAB2702364, 1:100), mouse anti-DNA Ligase IV (SCBT, #sc-271299, 1:50), and mouse anti-XRCC4 (SCBT, #sc-271087, 1:50). The Nissl staining was performed using NeuroTrace 435/455 Blue Fluorescent Nissl Stain (Invitrogen, #N21479, 1:700).

### Immunofluorescence (IF)

SH-SY5Y cells expressing TDP-43 WT or mNLS were cultured in 8-well chamber slides (Millicell EZ slides, Millipore), treated with DMSO or 10 μM etoposide (Sigma, #341205) for 2 h following a recovery step at pre-defined timepoints, fixed in 4% PFA in phosphate-buffered saline (PBS) for 20 min and permeabilized with 0.5% Triton X-100 in PBS for 20 min at room temperature. Blocking was performed with 3% BSA solution in PBS for 30 min. Then cells were incubated with primary antibodies overnight at 4 °C in 1% BSA solution and fluorescent secondary antibodies for 1 h at 37 °C. Slides were washed 3 times and counterstained with DAPI.

For IF on tissue samples, sections were blocked in 10% normal goat and/or donkey serum, 1% gelatin in 1x TBS-T for 30 min prior to primary antibody incubation. Alexa Fluor 488 and 568 conjugated antibodies raised in goat or donkey were used as secondaries (1:500, Molecular Probes, Invitrogen). Nuclei were counterstained with SlowFade^™^ Diamond Antifade Mountant with DAPI (Invitrogen, #S36964). Images were captured in AXIO Observer inverted fluorescence microscope (Carl Zeiss).

### Image analysis

Two sections each of the brain, spinal cord, and muscle sections were analyzed for each of the 6 animals of each genotype. Motor neurons were identified by their location in the cortex and ventral horn, as large nucleus, prominent nucleolus, polygonal shapes, and relatively large sizes. Only cells with clearly visible nucleolus were chosen for analysis. Images were captured using a Zeiss LSM 510 laser scanning microscope with a 63x oil immersion objective and 2x digital zoom. Image analysis was performed using ImageJ software (NIH). To quantify nuclear TDP-43, images of motor neuron nuclei were displayed in ImageJ with automatic setting of brightness and contrast. All distinct speckles of nuclear TDP-43 fluorescence with a minimum diameter of 1 μm were then counted manually using the point picker function. Cajal bodies and TDP-43 speckles were counted blind to genotype.

### Immunoblotting (IB) of brain tissues

Snap-frozen brain samples were ground by mortar and pestle using liquid nitrogen. Then approximately 20 mg powdered tissue samples were lysed in 200 μL of 1x RIPA lysis buffer added with cocktail protease and phosphatase inhibitors (Roche), centrifuged at 14000 rpm at 4 °C for 15 min 3-4 times until the white fat pellet was completely disappeared. Protein concentration was estimated by Bradford assay. About 20 μg of protein solutions were taken from each sample for WB analysis of γH2ax levels in sham versus ALS samples using anti-phospho-Histone H2AX and anti-GAPDH (Novus, #NB300-221, 1:2500) antibodies.

### Proximity ligation Assay (PLA)

Paraffin-embedded mouse brain and spinal cord tissue samples were de-paraffinized, antigen retrieved, and permeabilized in permeabilization buffer containing 1% Triton X-100 and 1% gelatin in 1x TBS-T buffer for 30 min at room temperature. *In situ* protein-protein interaction was analyzed using PLA (Duolink) kit, as per the manufacturer’s instructions [11]. Images were analyzed in AXIO Observer inverted microscope (Carl Zeiss).

### Thioflavin-S staining

Each tissue section was incubated in 500 μM of thioflavin-S (Sigma-Aldrich) solution, dissolved in 50% ethanol, for 7 min at room temperature, as described elsewhere [33]. Hoechst-33342 (10μg/mL, Sigma-Aldrich) was used to observe the nuclear morphology. Images were captured and analyzed in AXIO Observer inverted microscope (Carl Zeiss). The number and area of plaques detected by thioflavin-S were quantified using ImageJ software.

### Histopathology

Hematoxylin and Eosin (H&E; Abcam, #ab245880) and Congo Red (Abcam, #ab150663) staining procedures were performed on mouse brain, spinal cord, and soleus muscle tissue sections, following the manufacturer’s protocol.

### Senescence staining

The CellEvent^™^ Senescence Green Detection Kit (Invitrogen, C10850) was optimized at 1:800 for 3 h at 37 °C condition for mouse brain and spinal cord sections of 5 μm thickness. Neurons were identified by co-staining the slides with NeuroTrace Nissl staining reagent.

### TUNEL assay

The Click-iT^™^ Plus TUNEL Assay Kit (Invitrogen, #C10617) was used for *in situ* detection of DNA DSBs in the nuclear genome of cells from brain and spinal cord sections, according to the manufacturer’s instructions [11]. TUNEL images were taken under a brightfield microscope and analyzed by ImageJ software.

### Long amplicon PCR (LA-PCR) assay

Genomic DNA was isolated from sham and ALS mouse brain using DNeasy Blood and tissue kit (Qiagen) per the manufacturer′s directions. DNA was quantified using Quant-iT^™^ PicoGreen^™^ dsDNA Kit (Invitrogen, #P7589) [11]. The accumulation of DNA strand breaks was measured by PCR amplification of long amplicons using LongAmp Taq DNA polymerase (New England Biolabs, #M0323) and three pairs of primers amplifying distinct genomic regions of *POLB, NEUROD*, and *NANOG* genes, as described elsewhere [11, 34]. For LA-PCR assay, 20mg of tissue powder were used for each sample to extract high quality genomic DNA using genomic-tip 20/G kit per the manufacturer’s directions. The thermal cycling profile and DNA concentrations were optimized prior to setting up the actual reaction. 20 ng of genomic DNA were used for each sample for LA-PCR assay using the optimized thermal profile 94 °C for 30 sec (94 °C for 30 sec, 59 °C for 30 sec, 65 °C for 10 min) for 24 cycles and 65 °C for 10 min. Internal primer pair (forward: 5′-TATGGACCCCCATGAGGAACA-3′; reverse: 5′-AACCGTCGGCTAAAGACGTG-3′) was used for normalization of template DNA across the samples. PCR products were separated in agarose gel and visualized using Gel Doc XR+ (BIO-RAD) system. DNA amplicon bands were quantitated by dsDNA PicoGreen^™^ assay as mentioned earlier.

### Quantitative real-time PCR (qRT-PCR) assay

RNA was extracted from cortical tissue samples using the RNeasy kit (Qiagen) and cDNA was reversed transcribed using oligo(dT) primers and Superscript III (Invitrogen). qRT-PCR was performed using 5μM of each primer with Power SYBR Green master mix on an ABI Prism 7700 real-time PCR machine (Applied Biosystems). Expression levels of murine endothelin 1 (Edn1), p21, ankyrin 1 (Ankrd1), interleukin-6 (Il-6), and tumor necrosis factor α (Tnf-α) were measured using respective primer pairs, as described elsewhere [35] or designed as follows:

mAnkrd1-F: 5'-AGACTCCTTCAGCCAACATGATG-3'

mAnkrd1-R: 5'-CTCTCCATCTCTGAAATCCTCAGG-3'

mEDN1-F: 5'-GCACCGGAGCTGAGAATGG-3'

mEDN1-R: 5'-GTGGCAGAAGTAGACACACTC-3'

and normalized to the internal control murine Gapdh level. Relative expression levels were expressed using 2^−ΔΔCt^ method.

### Rotarod

Mice were trained for 3 days and tested following the procedures described elsewhere [36]. Briefly, the mice were placed on a rod (Ugo Basile Rota-Rod 47,600) rotating at 8 rpm constant speed. In the testing phase, the rotation speed was accelerated from 8 to 30 rpm in 5 min. Latency and fall-off rpm of each mouse was recorded when the mice fell from the rod.

### Hindlimb-clasping test

The mice were suspended by grasping their tails and their hindlimbs position were observed for 20 sec, as described previously [37]. The normal mice consistently kept their hindlimbs away from the abdomen. Hindlimbs of the ALS-Tdp-43ΔNLS mice exhibited abnormal movement in their left or right hindlimbs during the suspended time.

### DigiGait treadmill test

Locomotor performance was assessed using the DigiGait motorized transparent treadmill (Mouse Specifics, Inc.), which allows recording of animals from both a ventral and lateral view. Mice were pre-trained on the treadmill at speeds of 20 cm/sec and then tested weekly for analysis of the diseased state. Age-matching Ubc-Cre::Tdp-43ΔNLS mice were tested before and after the administration of TAM, whereas MN-specific Tdp-43ΔNLS mice were evaluated for their motor functions at 6- and 12-month of age. After a 2 min acclimatization to the treadmill, mice were recorded at a speed of 22 cm/sec for 10-15 sec in three consecutive trials with 2 min rest periods in between recordings.

### Statistical analysis

All statistical analyses were carried out using the GraphPad Prism 9.0 software. Data are expressed as mean ± standard deviation (SD). Statistical significance of the results was determined by a one-way ANOVA, unpaired t-test, Tukey, or Mann-Whitney U test as appropriate. A P-value of <0.05 was considered statistically significant.

## Results

### Loss of nuclear TDP-43 affects genomic stability in neuronal cells

**Fig. 1.**
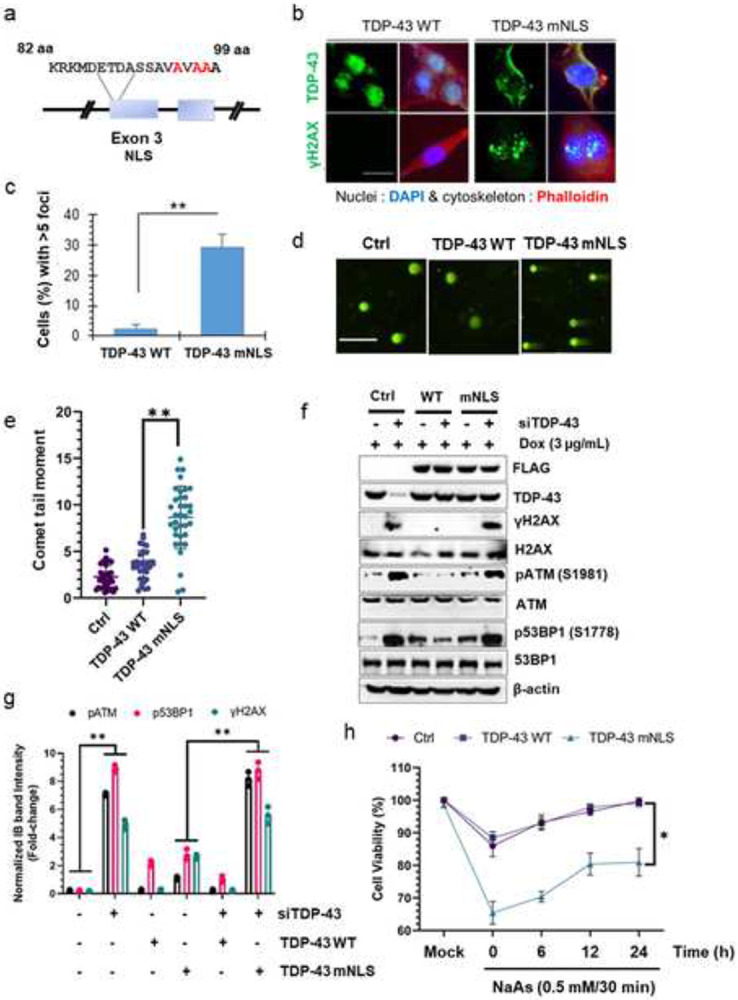
Inactivation of the TDP-43 nuclear localization signal (NLS) induces genomic instability. (***a***), Schematic presentation of NLS point mutations (K95A, K97A, and R98A) in TDP-43. (***b*** and ***c***), Immunofluorescence (IF) analysis of TDP-43 localization and DNA double-strand breaks (DSB) in doxycycline-inducible TDP-43 wild-type (WT) and mutationally inactivated NLS (mNLS) neuronal cells using anti-TDP-43 (*upper panel*) and anti-γH2AX antibodies (*lower panel*) (***b***). Nuclei were stained with DAPI and cytoskeleton with Alexa-Flour 568 Phalloidin. Scale bars, 10 μm. Quantitative analysis of γH2AX foci in the nucleus (n = 35 cells in each experiment) (***c***). Data were analyzed using a t-test from two independent experiments (N = 2); mean ± SD, **P < 0.01. (***d*** and ***e***), Neutral comet assay showing TDP-43 mNLS expression-induced DSB accumulation in neuronal cells compared to TDP-43 WT cells. Scale bars, 20 μm (***d***). Quantitation of comet tail moments for each experimental group (n = 50 cells); one-way ANOVA; **P < 0.01 (***e***). (***f*** and ***g***), doxycycline-induced WT or mNLS TDP-43 expressing neuronal cells were transfected with antisense RNA (siRNA) to the 3'UTR of *TARDBP* mRNA or control siRNA and harvested at 72 h post-transfection for immunoblotting (IB) analysis using indicated antibodies (***f***). Quantitation of IB band intensities is expressed in fold-change from two independent experiments (N = 2); one-way ANOVA; **P < 0.01 (**g**). (***h***) Cell viability was compared between the TDP-43 WT and mNLS expressing cells using the MTT assay upon exposing cells to the stress granule inducer sodium arsenite (NaAs) at 0.5 mM concentration for 30 min, followed by recovery at indicated time points. Data were analyzed by one-way ANOVA from two independent experiments (N = 2); mean ± SD, *P < 0.05.

Mimicking TDP-43 neuropathology in experimental models poses a dual challenge: the loss of nuclear TDP-43 indicating a loss-of-function (LOF) phenotype coupled with its accumulation in the cytosol, inducing a gain-of-toxicity (GOT) in impacted neurons [2, 38]. To attribute our previously reported DNA damage phenotype [11] to the loss of nuclear TDP-43, we generated a doxycycline-inducible SH-SY5Y cell model ectopically expressing either WT or NLS mutant human TDP-43 (hereafter referred to as hTDP-43-mNLS) (Fig. 1a). Dox induction (3 μg/mL) for 24 h showed robust nuclear clearance of flag-tagged hTDP-43-mNLS (Fig. 1b, *upper panel*, and Supplementary Fig. S1a) together with significant increase in γH2AX foci formation (Fig. 1b-c) compared to the WT TDP-43. We then compared the repair kinetics of DSBs in these cells induced by etoposide and observed more than two-fold increase in the number of γH2AX foci, together with significantly delayed DSB repair kinetics in the mNLS line compared to that in the control line (Fig. 1b-c and Supplementary Fig. S1b-c). We further validated higher baseline of genome damage in the mNLS line, using neutral comet assay to estimate endogenous DNA DSBs in these lines (Fig. 1d-e). Analysis of comet tail moments consistently revealed ~three-fold more unrepaired DSBs in the mNLS cells in the absence of any exogenous genome damaging agents (Fig. 1e). Further, we explored the status of DNA damage response (DDR) pathway in the WT versus mNLS expressing lines without any exogenous damage. Being an auto-regulatory protein, ectopically expressed TDP-43 is expected to maintain homeostasis of endogenous TDP-43 level [39]. Thus, we depleted the endogenous TDP-43 level by RNA interference (siTDP-43) in these cells to evaluate the genome-damaging effect of the mNLS variant solely in comparison to the WT variant. Immunoblotting (IB) results showed that levels of at least three classical DDR markers – γH2AX (S139), phospho-ATM (S1981), and phospho-53BP1 (S1778) – were significantly upregulated in siTDP-43-treated control and mNLS cells but not in WT TDP-43 expressing cells (Fig. 1f-g). Because overexpression of WT TDP-43 is toxic to cells [26, 40, 41], by downregulating the background TDP-43 level, we confirmed that the observed effects were primarily due to expression of the mNLS variant but not to its overexpression level. Consistent with previous findings [26], we also did not observe any significant difference in apoptosis rates between these two cell lines at baseline, however, acute promotion of nuclear clearance of WT and mNLS variants by NaAs (0.5 mM for 30 min) treatment [42] and a successive recovery phase of 24 h demonstrated a reduction in cell viability up to 20% in mNLS line compared to that in the WT or control line (Fig. 1h). These findings together with our previous reports suggest that both abnormal cytosolic accumulation as well as nuclear loss of functional TDP-43 collectively contributes to the DNA damage/repair imbalance, as observed in ALS/FTD patients, which prompted us to develop a conditional nuclei-cytosolic mislocalization mouse model not involving ectopic TDP-43 but by manipulating the endogenous murine Tdp-43 to better unravel the pathogenic mechanism of human ALS/FTD.

### Development of endogenous Tdp-43ΔNLS knock-in (KI) mouse model

**Fig. 2.**
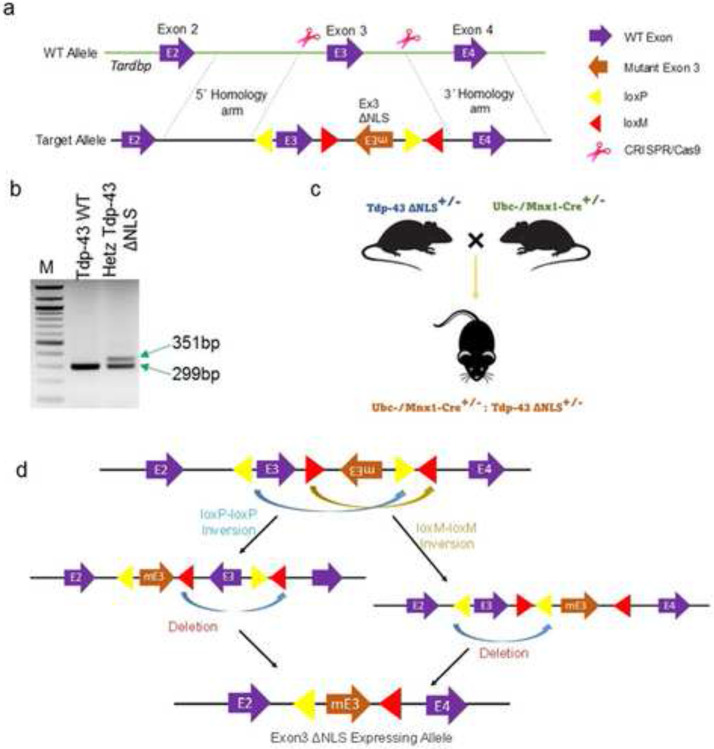
Generation of Tdp-43 knock-in mouse model. (**a**) Illustration of the target allele design for the murine *Tardbp* gene. (**b**) Genotyping PCR identifies a wild-type and a heterozygous littermate where the band size of 351 bp indicates the presence of the floxed target allele. (**c**) Schematic of the double-heterozygous Cre^+/−^:Tdp-43ΔNLS^+/−^ strain generation. (**d**) Illustration of the Cre-mediated recombination of floxed WT and mutant Exon-3 deletion and re-orientation, respectively, resulting in the expression of mutant Tdp-43ΔNLS variant in the desired cell type in the central nervous system (CNS).

In our continued efforts to mirror the mislocalization and collective effects of LOF and GOT of TDP-43, especially under non-overexpressing conditions, we embarked on the creation of a unique conditional CRISPR/Cas9-mediated KI mouse model expressing Tdp-43ΔNLS variant at the endogenous level. This model was designed to conditionally trigger the replacement of murine *Tardbp* Exon-3 with NLS-deleted mutant Exon-3 (mEx-3) through the FLEX Cre-switch strategy (Fig. 2b-c). In this cis-genic Tdp-43ΔNLS mouse model, the NLS-lacking mEx-3 flanking a converging pair of lox2272 (loxM) sequences was placed in reverse orientation (3′→5′), while the normal Ex-3 was flanked by a diverging pair of WT loxP sequences (Fig. 2a). We confirmed this genetic modification through genotyping with a pair of primers amplifying the 5' loxP sequence on the target allele (Fig. 2b). Next, we established the Tdp-43ΔNLS ALS model by crossing heterozygous Tdp-43ΔNLS^+/−^ mice with TAM-inducible Ubc (whole-body) or Mnx1 (MN-specific) promoter-driven Cre^+/−^ mice as shown in schematics (Fig. 2c-d). Thereby, we were able to generate two different types of murine Tdp-43ΔNLS mice models of ALS: one with inducible Cre-mediated Tdp-43ΔNLS expression and the other with developmentally regulated Mnx1-driven Cre expression-mediated Tdp-43ΔNLS in post-mitotic neurons in the brain. Given that homozygous Tdp-43 mutation renders the embryo non-viable [43], we used double-heterozygous animals for all experiments related to this study.

### Tdp-43ΔNLS expression induces mislocalization and aggregation pathology in the mice CNS

**Fig. 3.**
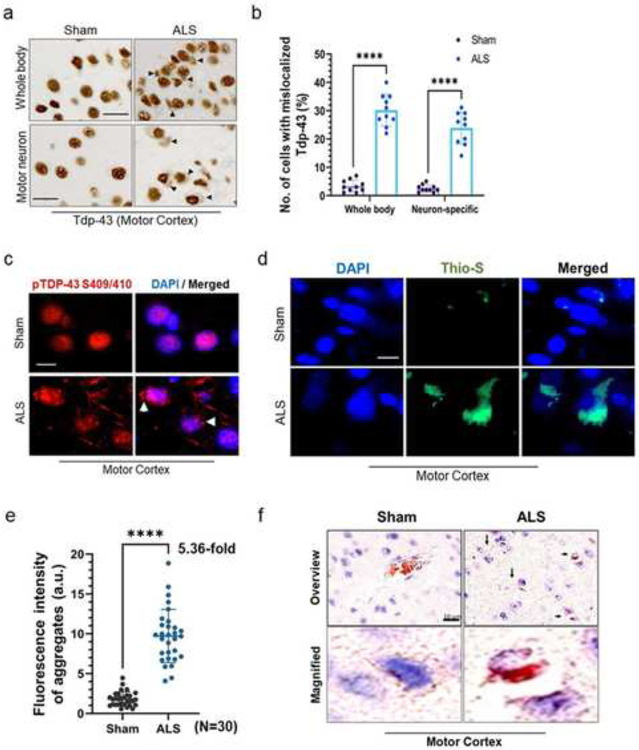
Neuronal Tdp-43ΔNLS expression induces Tdp-43 mislocalization and formation of pathological aggregates in the cytosol (**a-b**) Immunohistochemistry (IHC) with anti-TDP-43 antibody in the motor cortex of ALS mice Ubc-Cre::Tdp-43ΔNLS (whole body hence after; *upper panel*) and Mnx1-Cre::Tdp-43ΔNLS (neuron-specific hence after; *lower panel*). Scale bars, 20 μm (**a**). (**b**) Quantitation of the number of cells with Tdp-43 mislocalization phenotype in sham versus whole body or neuron-specific mice cortical tissues (N = 5 mice per group). (**c**) Representative IF images with anti-phosphoTdp-43 (S409/410) antibody in the motor cortex of sham versus whole body or neuron-ALS mice brains. Nuclei were counterstained with DAPI. Scale bars, 10 μm; N = 5 mice per group. (**d-e**) Thioflavin-S staining images of the cortical tissue from sham and ALS mice brains. Nuclei were counterstained with DAPI. Scale bars, 10 μm (**d**). (**e**) Quantitation of fluorescence intensity (arbitrary unit, a.u.) of Thioflavin-S-positive aggregates (N = 30 cells from cortical tissues of 5 mice in each group). (**f**) Representative Congo Red staining images of the cortex from sham and ALS mice. Pink stain indicates amyloid plaques in the cytosol and inter-cellular spaces. Nuclei were counterstained with hematoxylin. Scale bars, 10 μm. Data are expressed as mean ± SD and a nonparametric Mann-Whitney rank test was used for inter-group comparisons. ****P < 0.0001.

We examined the neuronal populations displaying murine Tdp-43 mislocalization in both the whole-body and MN-specific Tdp-43ΔNLS mice. IHC analysis of mouse brain cortical sections revealed an average of approximately 30% of brain cells with notable Tdp-43 mislocalization in the whole-body model, and approximately 25% in the MN-specific model in comparison to their respective sham mice controls (Fig. 3a-b). Given that TDP-43 proteinopathy initiates in the MN in ALS and FTD pathologies, from now onwards, we present histopathology and biochemical analysis results for the MN-specific Tdp-43ΔNLS mouse model as the ALS model unless stated otherwise in the following results. Next, we evaluated the extent of Tdp-43 protein aggregation in the realm of protein mislocalization in the CNS of mutant mice. Unlike in sham mice, the IF staining with anti-pTDP-43 (S409/410) antibody exhibited a significant number of distinct skein-like structures of hyperphosphorylated and mislocalized Tdp-43 in the cytosol of cortical neurons of mutant mice (Fig. 3c). Likewise, thioflavin-S staining indicated enhanced protein aggregation in the cytosol compared to that in age-matched sham mice brains (Fig. 3d-e). Congo red staining also confirmed the formation of amyloidogenic plaques in the nuclear periphery and/or cytosol in mutant mice cortices compared to that in sham mice (Fig. 3f). Next, we examined whether the motor neurons were predominantly affected by the Tdp-43ΔNLS expression.

**Fig. 4.**
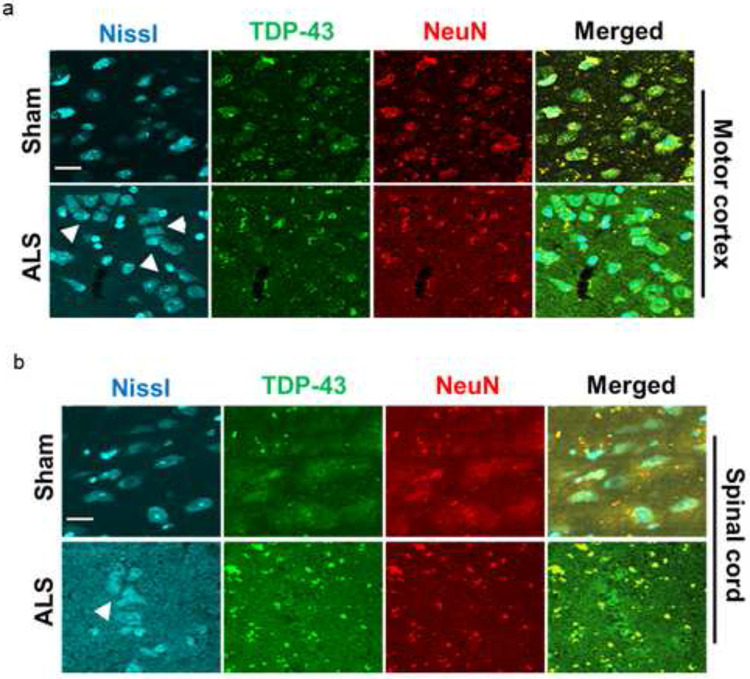
Tdp-43 mislocalization induces accumulation of NeuN transcription factor in the cytosolic protein aggregates (**a**) IHC staining with anti-TDP-43 and anti-NeuN antibodies in the motor cortex of ALS and sham mice. Scale bars, 10 μm. (**b**) IHC staining with anti-TDP-43 and anti-NeuN antibodies in the thoracic region of the spinal cord cortex of ALS and sham mice. Scale bars, 10 μm. Nuclei were counterstained with NeuroTrace Nissl staining (435/455 nm). White arrowheads in the Nissl-stained images indicate morphologically degenerating neurons in the motor cortex and ventral horn of the thoracic spinal cord.

Co-immunostaining of brain and spinal cord tissue sections with anti-TDP-43 and anti-NeuN revealed co-localization of both TDP-43 and NeuN in the cytosolic protein aggregates in the inner layers (III-V) of the motor cortex (Fig. 4a) and thoracic region of the spinal cord (Fig. 4b) of mutant mice but not in sham mice, suggesting that the observed ALS-like phenotypes in MN-specific Tdp-43ΔNLS mice were primarily due to Tdp-43 pathology in Mnx1-positive motor neurons in the CNS. In addition, the mutant mice group exhibited gradually increasing bodyweight from 3 months to 12 months of ages, however, marked variations in bodyweights were observed in mutant mice between 6 and 12 months of age compared to that in the sham group (Supplementary **Fig. S2),** possibly due to the Tdp-43 pathology-associated movement disorders, resulting in either obesity [44] or malnutrition.

### Tdp-43 mislocalization induces muscle atrophy and gait deformities

**Fig. 5.**
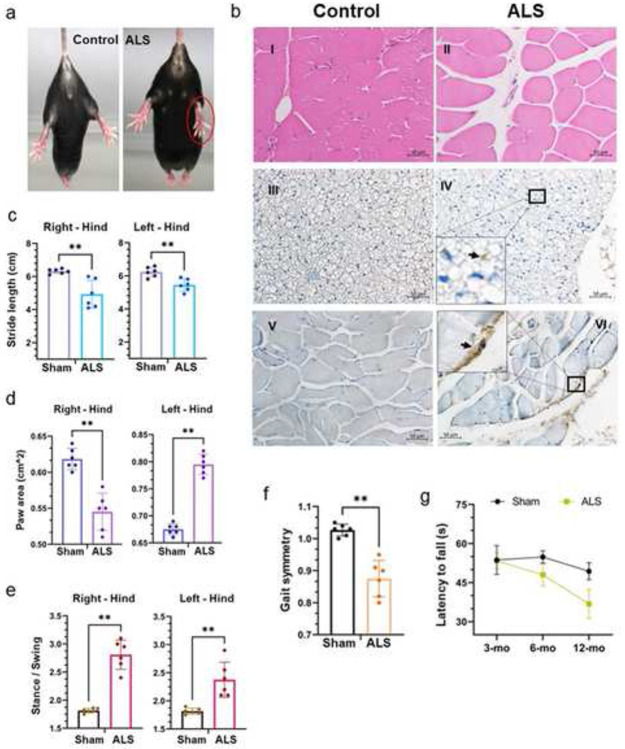
Motor neuron-specific Tdp-43ΔNLS expression causes muscle atrophy and gait deficits in Tdp-43 mutant mice. (**a**) Representative live-mice images show abnormal hindlimb reflexes in 12-month-old ALS-Tdp-43 mice but not in sham-control mice. (**b**) Histopathological analysis of sham and ALS mice soleus (I-II) and spinal muscle tissues (III-VI). Hematoxylin-Eosin (H&E) staining displays degenerating muscle disc ALS mice compared to the sham mice muscles (I-II). IHC staining with anti-phosphorylated TDP-43 (S409/410) revealed the presence of pathological pTDP-43 in the spinal skeletal muscle of ALS mice (III-IV) and soleus muscles (V-VI). Scale bars, 50 μm. Inset images show cytosolic pTDP-43 staining in muscle cells. (**c**) DigiGait analysis indicates reduced stride length of hindlimbs in ALS compared to that in sham mice. (**d**) Hindlimb paw area (cm^2^) measurements exhibited a decrease in paw area in the right hindlimb while an opposite effect was observed for the hind-left limb than that in sham mice. (**e**) Stance-to-swing ratios were higher for both hindlimbs in ALS mice than that in sham mice. (**f**) ALS mice presented reduced gait symmetry, overall, when compared to those in sham mice. (**g**) Rotarod testing showed a gradually decreasing trend in latency to fall for ALS mice compared to age-matched sham mice. Data are expressed as mean ± standard deviation (SD) and a nonparametric Mann-Whitney rank test was used for inter-group comparisons. **P < 0.01.

To assess the impact of Tdp-43 proteinopathy on motor functions, we initially conducted a tail suspension test to observe signs of limb clasping behavior in mutant and sham mice. Each mouse was suspended for 30 sec, and the test was repeated thrice. Unlike their WT counterparts, mutant mice exhibited an inability to stretch out their hind limbs normally (Fig. 5a). To investigate the health of muscle fibers, we performed histological analysis of soleus muscle sections from 12-month-old mutant and age-matched sham mice, revealing an abnormal distribution of satellite cells and irregular shapes of muscle fibers in the mutant mice using hematoxylin and eosin (H&E) staining, which showed irregular muscle fiber morphology and abnormal satellite cell distribution in mutant mice (Fig. 5b, I-II). Consistently, IHC with anti-pTDP-43 (S409/410) also indicated enhanced signal intensity in the spino-skeletal (Fig. 5b, *III-IV*) and soleus muscles (Fig. 5b, *V-VI*).

Subsequent evaluation of gait parameters revealed significant differences between the mutant and WT groups, with mutant mice displaying a considerable reduction in stride length (cm) of the hind limbs compared to sham mice, indicating development of significant gait abnormalities (Fig. 5c). Analysis of paw area (cm^2) showed a significant decrease in the right hind limbs of mutant mice relative to sham, with a reversed pattern observed in the left hind limbs (Fig. 5d). The stance to swing ratio was also notably higher in the hind limbs of mutant mice compared to that of sham controls (Fig. 5e). Likewise, the mutant mice group reflected a significantly lower gait symmetry than the sham group (Fig. 5f). Additionally, the rotarod test indicated a progressive decline in performance of latency as mutant mice aged from 3 months to 12 months (Fig. 5g). Notably, when we analyzed the paw angle and percent (%) Swing-Stride, we found left hind limb-centered gait defects in whole-body Tdp-43ΔNLS expressing mice (Supplementary **Fig. S3a-c),** while abnormal brake activity was significantly higher in the right hind limb of ALS (whole-body) mice than in sham mice (Supplementary **Fig. S3d**).

### Tdp-43 proteinopathy associates with neuronal genome damage in mice brain

**Fig. 6.**
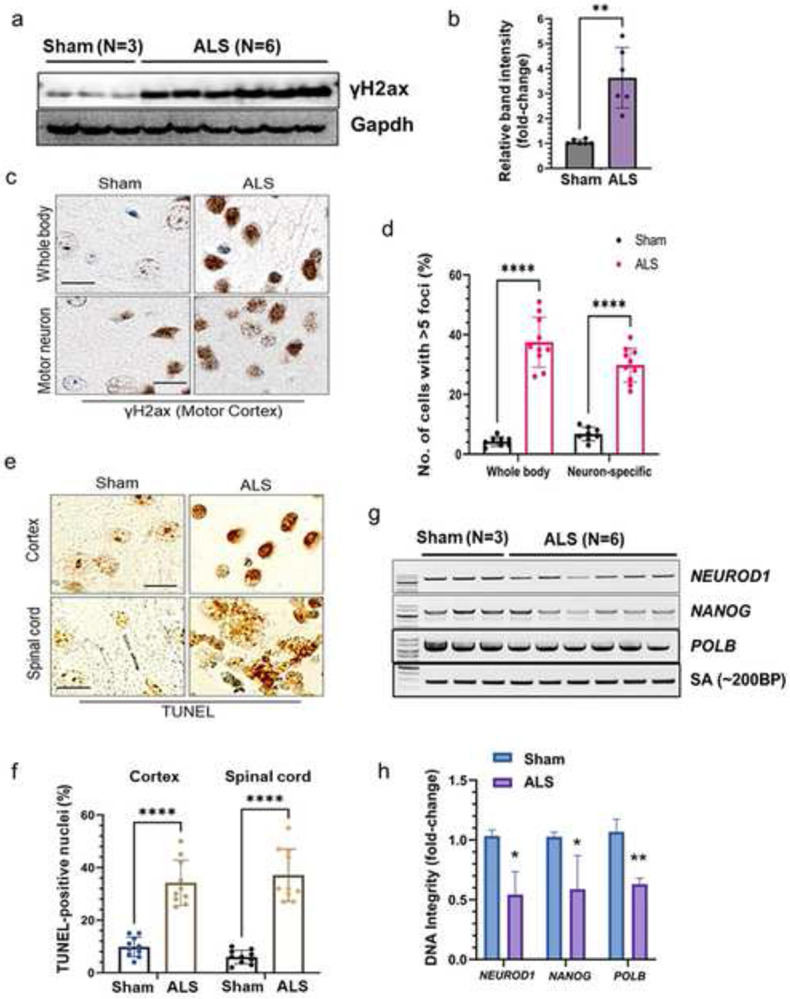
Tdp-43ΔNLS induces genome damage in the central nervous system (CNS). (**a-b**) Immunoblotting (IB) of cortical brain extracts from sham (N = 3) and whole boy Tdp-43 KI mice (N = 6) using anti-γH2ax. Gapdh served as the loading control (**a**). (**b**) Quantitation of relative band intensity of the ALS mice group compared to that of the sham mice group. (**c-d**) IHC analysis of γH2ax expressions in the cortex of the whole body and neuron-specific Tdp-43ΔNLS expressing mice. Nuclei were counterstained with hematoxylin (**c**). Scale bars: 20 μm. (**d**) Quantitation of the number of cells with >5 foci of γH2ax in the nucleus. (**e-f**) TUNEL analysis to estimate the neuronal genome damage in the cortex and spinal cord of neuron-specific Tdp-43ΔNLS expressing mice (**e**). (**f**) Quantitation of the number of cells with TUNEL-positive nuclei. Scale bars: 20 μm. (**g-h**) Long-range PCR amplification (LA-PCR) of ~10 kb length exhibited reduced genomic DNA integrity in the cortex of ALS mice (N = 6) compared to that in sham mice (N = 3). A 200 bp short-amplification product was used as an internal control (**g**). (**h**) Quantitation of relative band intensity of each genomic target in the ALS group than that in the sham group. Data are expressed as mean ± SD and analyzed by two-tailed nonparametric Mann-Whitney U test; mean ± SD; *P < 0.05, **P < 0.01, ****P < 0.0001.

Considering the pivotal role of TDP-43 in maintaining genomic integrity and given its mislocalization or nuclear clearance impairs DNA DSB repair in ALS-affected MNs [11], we examined the level of DSB marker γH2ax in cortical tissues of ALS and sham mice by western blotting which showed ~three-fold higher expression of γH2ax in mutant mice brain samples than in sham controls (Fig. 6a-b). Consistently, IHC analysis using anti-γH2ax antibody displayed significantly higher proportions of γH2ax-positive neuronal cells in the cortex of both the whole-body (*upper panel*) and MN-specific (*lower panel*) Tdp-43ΔNLS mice models than their respective WT sham mice (Fig. 6c-d). TUNEL analysis also confirmed significantly elevated levels of genome damage and TUNEL-positive cells in the cortex (*upper panel*) and spinal cord (*lower panel*) of mutant mice than in sham mice (Fig. 6e-f). Finally, we performed the long-range amplification PCR (LA-PCR) analysis to further corroborate that Tdp-43 mislocalization could inhibit an efficient repair of endogenously formed DSBs in the neuronal genome. Analysis of three distinct genomic regions of actively transcribing mouse genes *NEUROD, NANOG* and *POLB* using corresponding LA-PCR primer pairs demonstrated ~ two-fold reduced genome integrity in cortical samples of mutant mice than in sham mice samples (Fig. 6g-h).

### Mislocalized Tdp-43 traps selective DNA repair factors in the cytosol

**Fig. 7.**
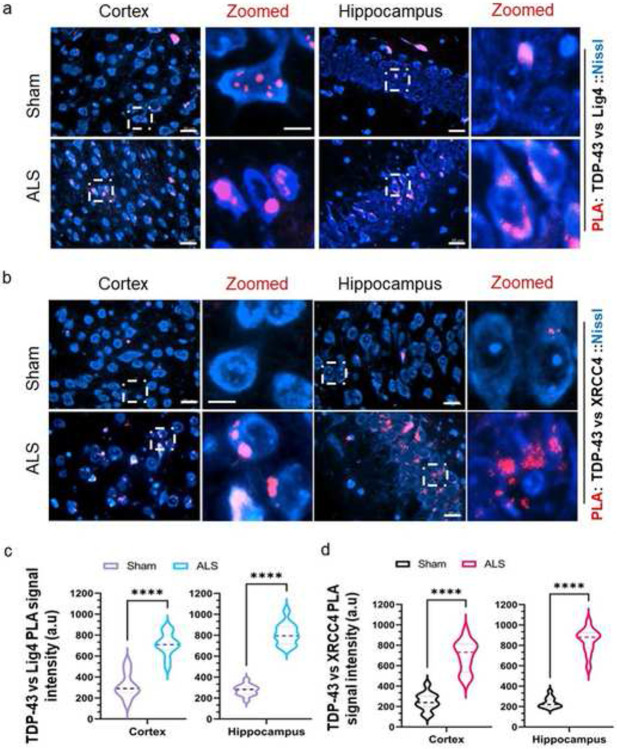
Tdp-43ΔNLS causes trapping of XRCC4 and Ligase 4 in the cytosol of neurons. (**a**) Representative images of proximity ligation assay (PLA) between TDP-43 and DNA Ligase 4 (Lig4) in the cortex and hippocampus of sham (upper panel) and ALS (lower panel) mice. Cell bodies were counterstained with Alexa-Fluor 488-conjugated Nissl stain. PLA signals were visualized as red foci/puncta at 568 nm. Scale bars, 10 μm (**b**) Representative PLA (TDP-43 vs. XRCC4) images in the cortex and hippocampus of sham (upper panel) and ALS (lower panel) mice. Scale bars, 10 μm Cell bodies were counterstained with fluorescent-tagged Nissl stain. (**c** and **d**) Quantitation of PLA signal intensity from 12 fields (two fields per animal) in each group for TDP-43 vs Lig4 (**c**) and TDP-43 vs XRCC4 (**d**). Data are expressed as mean ± SD and analyzed by two-tailed paired t-tests; ****P < 0.0001.

Aggregated or mislocalized TDP-43 sequesters and prevents the mobilization of the key DNA DSB ligation factors, XRCC4 and DNA Ligase 4 (Lig4), to the nucleus of human cells in response to nuclear genome damage [21]. We sought to explore if similar phenomena could be detected in this unique ALS-Tdp-43 mouse model. To do so, we performed PLA for anti-Tdp-43 versus anti-Lig4 or anti-Xrcc4 antibodies and counterstained neuronal cells with Nissl stain. We found that both in the cortex and hippocampus regions of mutant mice, TDP-43 interacted with Lig4 (Fig. 7a and c) or Xrcc4 (Fig. 7b and d) forming significantly intensified cytosolic puncta-like aggregated structures instead of clear foci as in the control samples. When we recapitulated the same assay between Tdp-43 and Lig4 in spinal cord samples of both mutant and sham mice, puncta were observed in the cytosol of ALS-affected neurons surrounding the nuclear periphery, unlike the sham mice (Supplementary **Fig. S4),** suggesting sequestering of Xrcc4 and DNA Lig4 with mislocalized Tdp-43 aggregates in the cytosol.

### Impaired DNA repair is associated with elevated neuroinflammation and accelerated neuronal senescence in Tdp-43ΔNLS mice

**Fig. 8.**
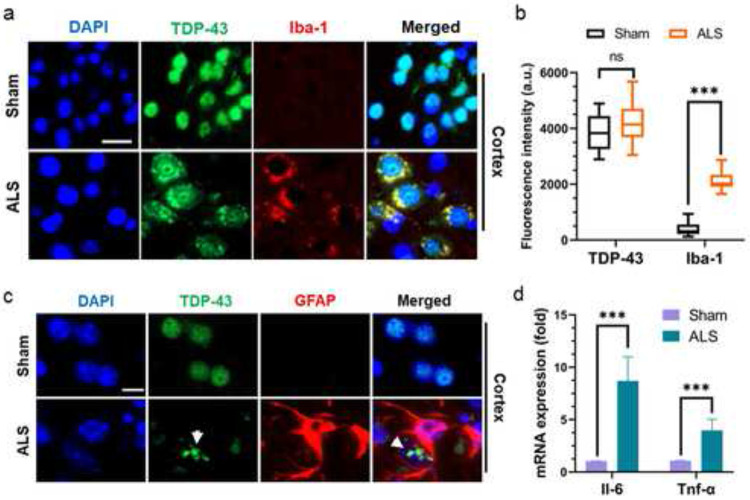
Tdp-43ΔNLS mice display signs of neuro-inflammation in the CNS. (**a-b**) Representative IF images showed enhanced expression of inflammatory marker Iba-1 in Tdp-43ΔNLS ALS mice’s cortex compared to sham mice (N = 6 /group) (**a**). Nuclei were counterstained with DAPI. Scale bars, 10 μm. (**b**) Quantitation of the signal intensity (a.u.). (**c**) Representative IF images displaying GFAP-positive astrocyte activation surrounding the cell with Tdp-43 aggregates (green; *indicated with a white arrowhead*) in the cortical region of ALS mice but not in sham mice. Nuclei were counterstained with DAPI. Scale bars, 10 μm. (**d**) Quantitation of relative mRNA levels (fold-change) of neuro-inflammatory markers Il-6 and Tnf-α in the cortical tissue of ALS and sham mice (N = 6). Gapdh served as the internal control. Data are expressed as mean ± SD and analyzed by two-tailed unpaired t-tests; ns = nonsignificant, ***P < 0.001.

Previous studies suggest that protein aggregation pathology induces hyperactivation of the neuro-inflammatory responses in the CNS of ALS patients [45, 46]. In this context, we evaluated the level of neuro-inflammatory marker Iba-1 expression in the motor cortex of mutant and sham mice. Co-localization analysis with anti-TDP-43 and anti-Iba-1 antibodies showed that neuronal cells with Tdp-43 mislocalization co-expressed Iba-1 at significantly higher levels in mutant mice cortices compared to that in sham mice cortices (Fig. 8a-b). No notable difference in Tdp-43 expression was observed between the groups. Moreover, co-staining with anti-Tdp-43 and anti-GFAP antibodies showed GFAP-positive differentiated astrocytes encircling neurons with Tdp-43 aggregates in mutant mice, a feature absents in sham controls (Fig. 8c).

**Fig. 9.**
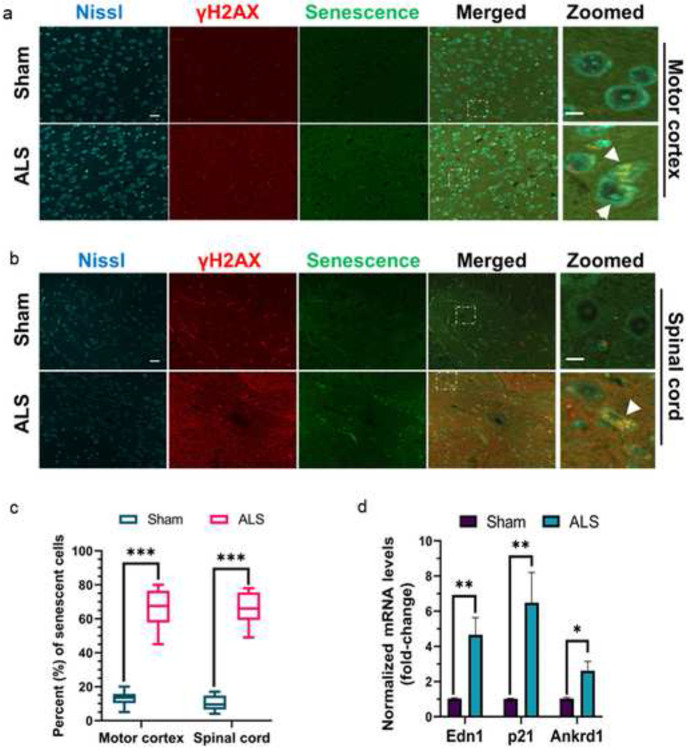
Tdp-43ΔNLS mice exhibit neuronal senescence phenotype in the CNS. (**a-c**) Representative IF with anti-γH2AX antibody combined with fluorescence-based senescence staining (CellEvent, Thermo) images showed higher populations of neurons dual-positive for γH2AX and senescence in the motor cortex (**a**) and spinal cord (thoracic) regions (**b**) of Tdp-43ΔNLS ALS mice than that in sham mice (N = 6 /group). Cell bodies were counterstained with Nissl. Scale bars, 40 μm (whole field); 10 μm (zoomed images). (**c**) Quantitation of the percent population of DNA damage-associated senescent cells. (**d**) Quantitation of relative mRNA levels (fold-change) of senescence-associated markers Edn1, p21, and Ankrd1 in the cortical tissue of ALS and sham mice (N = 6). Gapdh served as the internal control. Data are expressed as mean ± SD and analyzed by two-tailed unpaired t-tests; ns = nonsignificant, *P < 0.05, **P < 0.01, ***P < 0.001.

Quantitative RT-PCR analysis revealed significantly increased expressions of inflammatory markers such as Il-6 (~eight-fold) and Tnf-α (~four-fold) in the brains of mutant mice compared to sham controls (Fig. 8d). Notably, recent studies have linked the neuronal senescence phenotype to neuro-inflammatory conditions in several neurodegenerative diseases [47, 48]. Furthermore, DNA damage has been implicated in precipitating acute cellular senescence, independent of telomere shortening [49]. Hence, we sought to investigate the association of TDP-43 pathology-induced genome damage and neuronal senescence in this ALS-Tdp-43 mouse model. To identify neurons and their health conditions, we used fluorophore-tagged Nissl staining in tissue sections. As shown in Fig. 9, there were significantly higher populations of motor neurons with dual phenotypes of DNA damage (γH2ax-positive) and senescence in the inner layers of the motor cortex (~ four-fold higher; Fig. 9a and c) and thoracic region of the spinal cord (~four-fold; Fig. 9b and c) in the CNS of ALS-Tdp-43 mice than that in sham mice. Moreover, the mutant mice brain and spinal cord exhibited higher incidence of degenerating neurons, as marked by irregular shapes of the soma (indicated by white arrowheads) than their age-matched control counterparts. Additionally, qRT-PCR analysis of expressions of senescence-associated markers in the cortex of mouse brain showed significantly enhanced mRNA levels of Edn1 (~four-fold), p21 (~six-fold) and Ankrd1 (~three-fold) in mutant mice than in sham mice (Fig. 9d). Together, these results suggest Tdp-43ΔNLS expression in the MN may induce physiological DNA DSB repair inhibition, leading to persistent inflammation and senescence-mediated loss of neurons in the CNS in ALS.

## Discussion

TDP-43 mislocalization and aggregation are key features observed in a majority of ALS cases and approximately 40% of FTD patients [1, 50, 51]. The neuropathological and clinical findings indicate that overlapping pathogenic mechanisms involving TDP-43 proteinopathies contribute to neurodegeneration [52-54]. Nevertheless, reproducing human ALS/FTD symptoms in rodent models has been challenging due to TDP-43's complex regulation and sensitivity to dosage changes [39, 55].

In our initial cell model experiments, we observed that even partial nuclear clearance of TDP-43 was sufficient to disrupt the balance of endogenous DNA damage and repair, leading to accumulation of unrepaired DNA breaks in the nuclear genome. While Winton et al. 2008 [56] previously reported correlation of deletion of the NLS sequence in TDP-43 with its enhanced tendency for mislocalization and aggregation, our study is the first to establish the connection between TDP-43 mislocalization and spontaneous genome instability in neuronal cells. This phenomenon was more pronounced under conditions that induce DNA damage such as exposure to etoposide (a DNA topoisomerase-II poison), causing delayed repair of induced DNA breaks [57]. Consistently, neuronal cells expressing TDP-43-mNLS mutant were more vulnerable under oxidative stress conditions.

To further investigate ALS pathogenesis, we generated a novel endogenous CRISPR KI Tdp-43ΔNLS mouse model conditionally expressing a murine Tdp-43 variant lacking NLS (82-98 aa) sequence. This model uniquely mimics the early stages of ALS, showing TDP-43 mislocalization and progressive aggregation, the key pathological features of disease progression. Our model eliminates the limitations of previous models, such as rapid onset of unrelated motor symptoms and technical artifacts from constitutive transgene insertion.

We utilized the FLEX-based activation to target the *Tardbp* allele, employing Cre driver-mediated recombination of loxP-loxP or mutant loxM-loxM sites. This design ensures control over the recombinase reaction in differentiated or mature cell types, preventing Cre-loxP-mediated aberrant chromosomal rearrangements and loss of the target allele in embryonic stem cells [58]. Our endogenous KI model is thus novel and distinct from previous ALS-TDP-43 overexpression and downregulation mice models [27, 43, 44, 59-61], and NLS-deleted Tdp-43 variant from the murine *Tardbp* gene’s locus is expressed, specifically in motor neurons. This approach eliminates the potential complications of transgene insertion and the development of aggressive motor phenotypes, which do not mimic human ALS pathophysiology.

To recapitulate the disease progression from pre-symptomatic to symptomatic stages, we incorporated a hemizygous bigenic MN-specific Tdp-43ΔNLS allele in most of the studies. The bigenic Cre::Tdp-43ΔNLS mice suffered from progressive motor dysfunctions, gait asymmetry, early-stage myogenic ALS pathology, and MN degeneration, simultaneously, pTDP- 43- and ubiquitin- positive pathology in the dorso-lateral and dorso-ventral spinal cord, reflected early ALS symptoms [62]. This progression is more nuanced compared to milder phenotypes of neuromuscular abnormalities seen in mice with mutations in Fus, VCP, and Sod1 [63-65]. In around 12 months of age, these mice progressively developed signs of gait disorders in their hind limbs, without paralysis or premature death. Notably, some males in this group experienced excessive weight gain potentially linked to Tdp-43 proteinopathy-induced abnormal fat metabolism [66], which may contribute to the observed gait disturbances.

Furthermore, our model is the first to demonstrate the link between Tdp-43 pathology accumulation of DNA break accumulation, as well as an enhanced neuroinflammatory response in both the brain and spinal cord. These disease phenotypes, while shown in various *in vitro* models by us [11, 21] and others [18, 20], have not been comprehensively explored in previous ALS models. Using a combination of cellular, molecular, and histopathological readouts, along with *in vivo* motor function tests, we demonstrate here that aberrant mislocalization of Tdp-43ΔNLS and its subsequent aggregation recapitulate the key pathologic features of ALS-TDP-43. Our analysis also revealed a potential crosstalk between proteinopathy, genome damage, neuroinflammation, and neuronal senescence in this early symptomatic ALS model.

While accumulating evidence underscores a critical connection between genome damage and neuron loss in ALS/FTD-TDP-43 and related diseases, to date, only rNLS8 (hTDP-43ΔNLS transgenic) line has shown approximately 2-3 folds overexpression of DNA-damage inducible transcript 3 (Chop), growth arrest, and DNA-damage-inducible 45 gamma (Gadd45γ), as the early markers of cellular stress and death [67-69]. However, the perturbed DNA repair and DNA damage response pathways were not tested in ALS-afflicted neurons. Hence, we tested this in our endogenous ALS-Tdp-43ΔNLS mouse model, and were the first to demonstrate in the brain (cortex and hippocampus) and spinal cord that nuclear clearance and subsequent pathological aggregation of Tdp-43 in MN-specific manner led to accumulation of DNA break foci, hyperactivation of neuroinflammatory factors such as Iba-1, Il-6, and Tnf-α, resulting in neuronal death. We also recapitulated our initial finding that aggregated mutant TDP-43 can trap DNA repair factors in the cytosol [21], thereby preventing their nuclear translocation in response to genome damage and inhibiting DNA repair processes including DNA DSB repair. Notably, although GFAP-positive astrocytes were activated or accumulated in the vicinity of damaged neurons (with DNA DSBs and protein aggregation), they did not exhibit any stress phenotypes, indicating that observed motor phenotypes were specifically caused by MN-specific Tdp-43 proteinopathy in these Mnx1-Cre::Tdp-43ΔNLS mice. Detailed investigations are critical for exploring impaired DNA repair in isolated cortical MNs from adult ALS mouse brains in order to develop DNA repair-targeted therapies for early-stage ALS patients. Thus, our unique ALS-Tdp-43ΔNLS mouse model is poised to serve as an ideal drug screening model.

NeuN, encoded by *FOX-3* gene, is an important neuron-specific transcription factor as well as RNA splicing regulator [70, 71]. The NeuN-synapsin-1 interaction plays a pivotal role in synaptic plasticity and regulation of levels of inhibitory neurotransmitters at the synapse through synaptic vesicles [72, 73]. Interestingly, brain trauma can induce TDP-43 mislocalization, along with altered localization of NeuN from the nucleus to the cytosol in the ipsilateral region of the cortex compared to the contralateral region of a non-transgenic mouse model [74]. Similar altered subcellular localization of NeuN has also been reported in HIV-associated neurocognitive disorders [75]. Notably, we also found that Tdp-43 mislocalization markedly altered the localization and sequestration of murine NeuN in the cytosol of stressed and degenerative neurons (clustered) [76] in the inner layers (III-V) of the motor cortex and ventral horn of the thoracic spinal cord of mutant mice via an unknown mechanism. Further investigation is needed to test whether NeuN, an RNA splicing factor, interacts with TDP-43 directly within a spliceosome.

Our findings also underscore the potential role of neuronal senescence in ALS/FTD pathogenesis. We found that senescent neurons were positive for DNA DSB marker γH2ax and a senescence probe, suggesting that loss of Tdp-43 function-linked DNA DSB accumulation might be a critical driver of neuronal senescence and cell death in ALS-TDP-43. This observation opens new avenues for understanding the relationship between cellular senescence, neuroinflammation, and neurodegenerative diseases. Although early senescence may confer protection to cells against lethal damage [77-79], emerging evidence suggest that neuronal senescence plays a pivotal role in neuron loss, resulting in the motor and cognitive dysfunctions in ALS/FTD [80-82]. Furthermore, there is an important crosstalk between cellular senescence and neuroinflammation. C-X-C motif chemokine receptor 2 (CXCR2) was found to increase significantly triggering neuronal apoptosis in sporadic ALS [83]. On the other hand, senescent cells activate CXCR2-mediated self-amplifying secretory network which promotes growth arrest [84]. Consistently, we demonstrate that senescent cells were Nissl-positive and γH2ax-positive, indicating damage-associated senescence in neuronal cells in MN-specific Tdp-43ΔNLS mice brain and spinal cord. Future research should focus on dissecting the molecular characteristics of these senescent motor neurons and exploring DNA repair-targeted therapies for early-stage ALS. Our unique ALS-Tdp-43ΔNLS mouse model provides an ideal platform for such investigations, potentially advancing our understanding of ALS and FTD and informing the development of novel therapeutic strategies.

Our ALS-Tdp-43 mouse model demonstrates the clear manifestation of key pathological hallmarks of ALS/FTD at the molecular level, while maintaining a non-paralytic motor deficit condition. As such, it can offer investigators unique opportunities to decipher the disease-causing early-stage pathogenic mechanisms in MNs that might be reverted by therapeutic drugs, even in long-term treatments – a condition that is difficult sustain in other aggressive disease models.

In conclusion, multiple animal models for ALS/FTD are obligatory for a comprehensive understanding of their complex and progressive pathogenesis. Such models, including those based on overexpression or knockdown of critical genes, play a crucial role in unraveling specific etiological functions and/or toxicity of the disease-related proteins. Our model, uniquely replicating both the nuclear loss of Tdp-43 and its cytosolic aggregation – two hallmark features of ALS/FTD – adds a new dimension to the existing array of animal models. This model not only mirrors key disease mechanisms, including protein mislocalization, aggregation, and markers of TDP-43's pathological forms but also genome instability, inflammation, senescence, and neuronal dysfunction, along with gait abnormalities. Furthermore, this model offers the opportunity to investigate the early-stage etiological factors of ALS and the concomitant impact of aging on ALS progression. Such comprehensive representation will make this model an invaluable tool for testing new therapeutic concepts and deepening our understanding of these debilitating neurodegenerative diseases.

## Data Availability

All relevant data generated and analyzed in this study are available in this manuscript, online supplementary information or upon reasonable request.
